# Agnoprotein Is an Essential Egress Factor during BK Polyomavirus Infection

**DOI:** 10.3390/ijms19030902

**Published:** 2018-03-19

**Authors:** Margarita-Maria Panou, Emma L. Prescott, Daniel L. Hurdiss, Gemma Swinscoe, Michael Hollinshead, Laura G. Caller, Ethan L. Morgan, Louisa Carlisle, Marietta Müller, Michelle Antoni, David Kealy, Neil A. Ranson, Colin M. Crump, Andrew Macdonald

**Affiliations:** 1 Faculty of Biological Sciences and Astbury Centre for Structural and Molecular Biology, University of Leeds, Leeds LS2 9JT, UK; bs13mmp@leeds.ac.uk (M.-M.P.); e.l.prescott@leeds.ac.uk (E.L.P.); bs10d2h@leeds.ac.uk (D.L.H.); ed10ges@leeds.ac.uk (G.S.); bs10elm@leeds.ac.uk (E.L.M.); m.muller@leeds.ac.uk (M.M.); bsman@leeds.ac.uk (M.A.); bsdjk@leeds.ac.uk (D.K.); n.a.ranson@leeds.ac.uk (N.A.R.); 2 Department of Pathology, University of Cambridge, Tennis Court Road, Cambridge CB2 1QP, UK; msh57@cam.ac.uk (M.H.); lgw27@cam.ac.uk (L.G.C.); c.w.wasson@leeds.ac.uk (L.C.); cmc56@cam.ac.uk (C.M.C.)

**Keywords:** polyomavirus, agnoprotein, virus exit

## Abstract

BK polyomavirus (BKPyV; hereafter referred to as BK) causes a lifelong chronic infection and is associated with debilitating disease in kidney transplant recipients. Despite its importance, aspects of the virus life cycle remain poorly understood. In addition to the structural proteins, the late region of the BK genome encodes for an auxiliary protein called agnoprotein. Studies on other polyomavirus agnoproteins have suggested that the protein may contribute to virion infectivity. Here, we demonstrate an essential role for agnoprotein in BK virus release. Viruses lacking agnoprotein fail to release from host cells and do not propagate to wild-type levels. Despite this, agnoprotein is not essential for virion infectivity or morphogenesis. Instead, agnoprotein expression correlates with nuclear egress of BK virions. We demonstrate that the agnoprotein binding partner α-soluble N-ethylmaleimide sensitive fusion (NSF) attachment protein (α-SNAP) is necessary for BK virion release, and siRNA knockdown of α-SNAP prevents nuclear release of wild-type BK virions. These data highlight a novel role for agnoprotein and begin to reveal the mechanism by which polyomaviruses leave an infected cell.

## 1. Introduction

Polyomaviruses are small, non-enveloped viruses that use mammals, fish and birds as their hosts [1,2,3,4]. Currently, thirteen human polyomaviruses have been discovered and a number are linked to disease [5,6,7]. The first two human polyomaviruses discovered, BK polyomavirus and JC polyomavirus (JCPyV; hereafter referred to as JC), were named after the index case patients upon their discovery more than 40 years ago [8,9] and cause disease in immunosuppressed patients. JC is the causative agent of the lethal brain disease progressive multifocal leukoencephalopathy.

BK is an opportunistic pathogen, and is associated with several diseases in the immunosuppressed [10]. Primary infection with BK typically occurs in childhood, after which the virus establishes a chronic infection in the kidneys in approximately 80% of adults [11]. Whilst reactivation of BK does occur in healthy individuals, this is usually associated with asymptomatic low-level urinary shedding [12]. However, in the immunosuppressed, reactivation of BK is far more serious, resulting in increased urinary shedding because of increased replication in the absence of a competent immune response [13,14]. Such uncontrolled replication is ultimately linked with severe health problems, including polyomavirus-associated nephropathy (PVAN) and hemorrhagic cystitis in kidney and bone marrow transplant patients, respectively [15,16]. Up to 10% of kidney transplant patients experience PVAN, and of these, up to 90% may go on to lose their graft [17]. The incidence of BK-associated disease is rising due to the increase in transplants, and the use of more powerful immunosuppressive drugs to support such patients [11]. Despite the clinical impact of BK-associated disease, no anti-viral drugs that specifically target BK, or indeed any human polyomavirus, are currently available. Rather, generic anti-viral agents such as Cidofovir can be used, however, these have low efficacy and are themselves associated with nephrotoxicity [18]. Treatment is typically limited to a reduction in immunosuppression, which runs the risk of transplant rejection [19]. A better understanding of the BK life cycle is therefore needed in order to identify new targets for anti-viral therapy.

BK and JC polyomaviruses are closely related to the prototypic primate polyomavirus simian vacuolating agent 40 (SV40) [1]. Their ~5000 bp double-stranded DNA (dsDNA) genome is divided into three functional units consisting of early and late coding regions, separated by a non-coding control region (NCCR) [6]. The NCCR contains the origin of virus replication as well as enhancer and regulatory regions that control virus transcription. In kidney transplant recipients, circulating BK strains undergo re-arrangement of the NCCR region and this is thought to play an important role in disease [20]. The early region encodes the small (sT) and large (LT) tumor antigens, essential for virus transcription and replication. The late region encodes for the major (VP1) and minor capsid (VP2/VP3) proteins, which form the structural components of the BK virion [4,21], as well as the non-structural auxiliary agnoprotein.

Agnoprotein is a small, highly basic protein encoded by only a minority of polyomaviruses [22]. Whilst none of the recently discovered human polyomaviruses encode an agnoprotein, their remains a striking diversity of agnoprotein sequence and size within the mammalian polyomaviruses containing an agnoprotein open reading frame [1,22]. Amongst this diversity, the agnoproteins of BK, JC and SV40 share a high degree of sequence identity, particularly within the amino-terminal half of the protein (up to 83% identity between BK and JC), suggesting a conservation of function. Agnoprotein is predominantly expressed within the cytoplasm and perinuclear regions of infected cells during the later stages of the polyomavirus life cycle [23]. More recently, agnoprotein has also been shown to co-localise with lipid droplets in BK infected primary renal tubular epithelial cells [24], however, the physiological relevance of this is currently unclear. The agnoproteins of BK, JC and SV40 are phosphorylated when expressed in cells, and studies have shown that this phosphorylation plays a critical role in the respective virus life cycle [22,23,25,26]. Despite these observations, mechanistic insight into the role of agnoprotein phosphorylation is lacking.

Whilst the precise function of BK agnoprotein is currently not known, studies in JC and SV40 have produced contradictory findings [22,27]. A number of studies have shown that changes in agnoprotein expression, either from deletion of the open reading frame (ORF) or mutation of its start codon, impact on expression of other virus proteins [28,29,30,31,32]. Given the abundant expression of agnoprotein at the later stages of the polyomavirus life cycle, a role in virion assembly, morphogenesis and release has also been suggested. In SV40, agnoprotein expression might be required for correct localization of the VP1 major capsid protein [33], and cells infected with SV40 virus lacking agnoprotein release progeny virions deficient in DNA content [34,35]. Similar findings have been reported for JC virus [35], however, loss of agnoprotein has also been correlated with a defect in virus release [28]. Studies using clinical isolates of BK virus containing deletions within the agnogene indicate that agnoprotein expression correlates with virion infectivity [36]. The reasons for such wide-ranging phenotypes associated with agnoprotein deficiency remain unclear.

In this study we aimed to increase our understanding of the role of this enigmatic protein in the BK life cycle by generating a mutation in the start codon of the agnogene in the disease-associated Dunlop strain of BK virus. Using a primary renal proximal tubular epithelial cell culture model system, we found that loss of agnoprotein led to a profound reduction in virion release and impaired virus propagation in culture. In contrast with previous findings we show that these virions are infectious but remain trapped within the nucleus of an infected cell. We implicate an agnoprotein binding partner, α-SNAP, as an essential BK egress factor. Together, these data demonstrate that agnoprotein is required for the release of infectious BK virions.

## 2. Results

### 2.1. Loss of Agnoprotein Increases BK Transcription and Protein Expression

**Figure 1:**
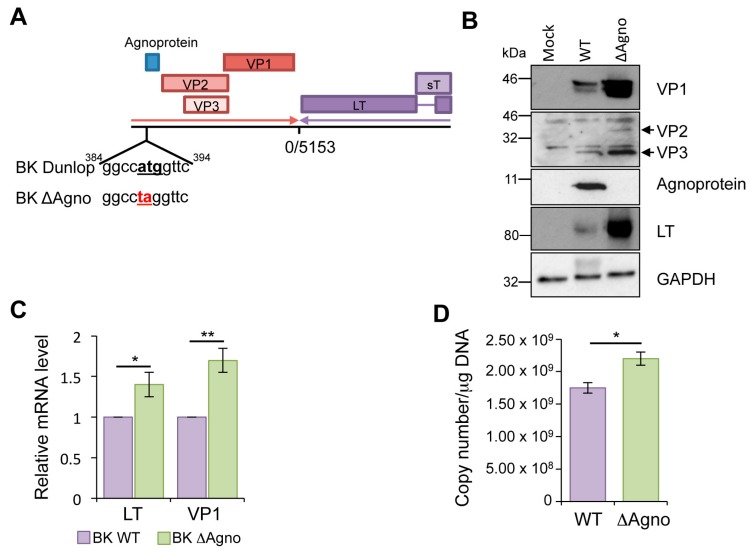
Loss of agnoprotein increases BK gene expression. (**A**) Schematic illustration of the BK Dunlop genome including the agnoprotein sequence mutated to generate the ΔAgno virus. Agnoprotein start codon in bold and base changes underlined in red; (**B**) Lysates from RPTE cells transfected with BK WT and ΔAgno genomes were probed with antibodies against early (LT) and late (VP1-3 and agnoprotein) proteins. Glyceraldehyde 3-phosphate dehydrogenase (GAPDH) was included as a protein loading control. Loss of agnoprotein correlated with increased expression of other virus protein products; (**C**) Levels of early (LT) and late (VP1) mRNA transcripts were measured from RPTE cells containing BK WT or ΔAgno genomes. Levels of virus transcript were increased in the absence of agnoprotein; (**D**) Virus genome replication was measured by qPCR in RPTE cells containing BK WT and ΔAgno virus. Genome replication was increased in the absence of agnoprotein. All experiments are representative of at least three independent experimental repeats. Significance of changes were analyzed by Student’s *t*-test and indicated by * *p* <0.05, ** *p* <0.01.

Agnoprotein is thought to be essential at several stages in the polyomavirus life cycle. To investigate this, we generated an agnoprotein knockout mutant in the clinically relevant Dunlop strain of BK. In this ΔAgno mutant, site directed mutagenesis was employed to replace the start codon (ATG) with a stop codon (TAG) (Figure 1A). Sequencing of the entire Dunlop genome confirmed the introduction of the mutation and established that no secondary mutations had been introduced. Equal amounts of wild type (WT) and ΔAgno genomes were transfected into primary renal proximal tubular epithelial (RPTE) cells, a physiologically relevant cell model for BK infection, and levels of BK protein expression determined at 72 h post transfection. Western blot analysis demonstrated production of early (LT) and late (VP1, VP2/VP3) proteins from both BK WT and ΔAgno genomes, and as expected only BK WT produced agnoprotein (Figure 1B). Interestingly, ΔAgno exhibited a consistent increase in virus protein expression compared to WT. Quantitative reverse transcriptase PCR (qRT-PCR) was used to determine if the increased BK protein expression was due to changes in virus gene transcription. Primer sets were used to amplify LT to detect early transcripts and VP1 to detect late transcripts. Levels of both transcripts were higher in ΔAgno transfected RPTE cells compared to WT BK control, suggesting that loss of agnoprotein correlates with an increase in early and late BK transcription. Given the role of LT in virus genome replication, we reasoned that increased expression of LT might result in increased virus replication. Indeed, in the absence of agnoprotein, levels of virus genome were higher than WT BK. Together, these data suggest that agnoprotein might play a role in the negative regulation of virus transcription and genome replication.

### 2.2. Agnoprotein Is Required for BK Virus Release

**Figure 2:**
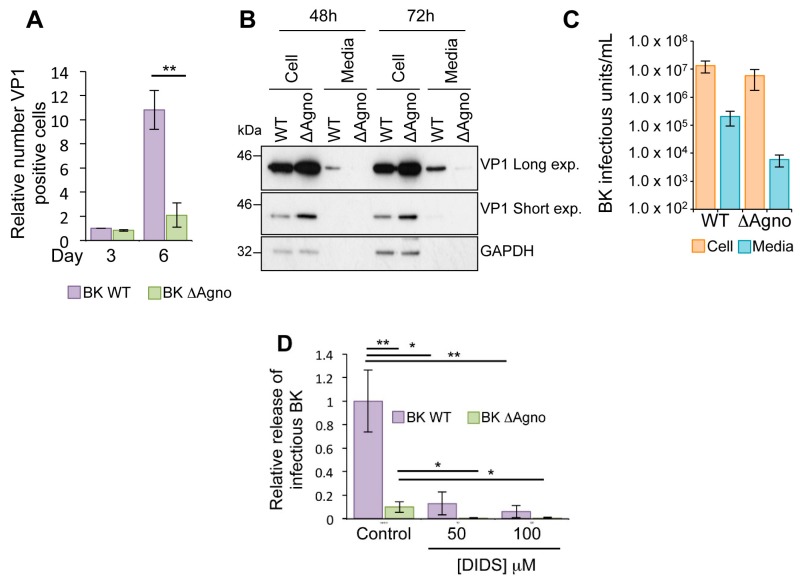
Agnoprotein facilitates virion release and enhances virus propagation. (**A**) RPTE cells transfected with BK WT and ΔAgno genomes were incubated over a 6-day time course, and levels of VP1 protein expression determined by indirect immunofluorescence using Incucyte Zoom software (Essen BioScience, Ann Arbor, MI, USA). Levels of VP1 expression are shown relative to the Day 3 BK WT sample. Significance of the changes were analyzed by Student’s *t*-test and indicated by ** *p* <0.01; (**B**) BK virus lacking agnoprotein fails to release virus into the cell culture media. Whole cell lysates and media samples from RPTE cells transfected with BK WT or ΔAgno genomes were analyzed at 48 and 72 h post-transfection for the VP1 capsid protein. GAPDH served as a protein loading control for the whole cell lysates; (**C**) RPTE cells were infected with BK WT and ΔAgno and cell-associated and media fractions harvested separately. Fluorescence focus assay was then performed to determine the IU/mL^−1^ of virus in the cells and supernatant; (**D**) Effect of the anion channel blocker DIDS is independent of agnoprotein. RPTE cells were infected with BK WT or ΔAgno and treated with dimethyl sulphoxide (DMSO) only (control) or 50–100 μM DIDS at 48 h post infection. Media and cell-associated fractions were harvested separately at 72 h post infection. Infectious virus titers were quantified by fluorescence focus assay on naïve RPTE cells and the proportion of total infectivity released into the media for each condition was calculated. Levels of released infectivity are represented as relative to the untreated BK WT samples. The graph corresponds to an average of three experimental repeats. Significance was analyzed by Student’s *t*-test and is indicated by an asterix * *p* <0.05, ** *p* <0.01.

To further investigate the role of agnoprotein, we performed a virus growth assay. RPTE cells were transfected with WT BK or ΔAgno genomes and the number of VP1 capsid protein positive cells determined using Incucyte Zoom software (Essen BioScience, Ann Arbor, MI, USA) [37]. Whilst numbers of VP1 positive cells were similar at three days post transfection, the number of VP1 positive cells in ΔAgno transfected cells was significantly lower at six days post transfection, suggesting that virus dissemination was impaired in the absence of agnoprotein (Figure 2A). Levels of VP1 were then measured from harvested cells and culture media supernatant at 48 and 72 h time points (Figure 2B). In agreement with our previous observations, VP1 levels were higher in the cell lysate of ΔAgno transfected RPTE cells compared to WT BK (Figure 2B). Low levels of VP1 protein were also detectable by Western blot in the media supernatant of WT BK transfected RPTE cells 48 h after transfection, and levels increased at the 72 h time point. In contrast, VP1 was undetected at 48 h in the supernatants of cells transfected with ΔAgno, and remained lower than the WT at the 72 h time point (Figure 2B). To rule out potential non-specific effects of transfection, RPTE cells were infected with 1 IU/cell WT and ΔAgno viruses and incubated for 72 h, and the cell lysate and culture media harvested separately. The infectious virus titer from each fraction was then determined by fluorescent focus assay (Figure 2C). Whilst there was a small decrease in cell-associated infectious virus from ΔAgno infected cells, the proportion of virus released was approximately 10 fold reduced (Figure 2C). Recently, the broad-spectrum anion channel inhibitor 4,4′-Diisothiocyanatostilbene-2,2′-disulphonate (DIDS) has been shown to impair the release of BK virus particles from RPTE cells [38]. Whilst the molecular basis by which DIDS prevents BK release is currently not known, DIDS has been shown to prevent enterovirus 71 (EV71) release by targeting the virus encoded 2B protein [39]. EV71 2B is a small hydrophobic protein belonging to the viroporin family of membrane permeabilizing proteins [40,41]. Given that JC agnoprotein has been described as a viroporin, we sought to determine whether BK agnoprotein might be the target for the inhibitory activity of DIDS. To investigate this, RPTE cells were infected with WT BK or ΔAgno, and DIDS added to cells 48 h post infection. At 72 h post infection cell-associated and culture media supernatant samples was harvested separately and used to infect fresh RPTE cells, from which the infectious titer of cell-associated and released BK virus was determined by a fluorescence focus assay [38]. Incubation with 50 μM DIDS resulted in an approximately ten-fold decrease in the proportion of released WT BK virus (Figure 2D). Increasing the dose of DIDS to 100 μM further reduced the proportion of released virus. The proportion of released virus from ΔAgno infected cells was 10-fold lower than WT BK control, and this was reduced further after DIDS treatment, in a concentration dependent manner. Together, these data show that agnoprotein is important for BK virus release, although via a pathway that is independent of the target of the DIDS compound.

### 2.3. Agnoprotein Is Not Required for the Production of BK Virions

**Figure 3:**
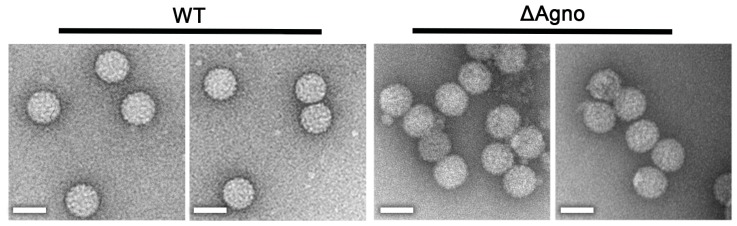
Loss of agnoprotein does not impair BK virion assembly. Negative stain electron micrograph of BK WT and ΔAgno virions following centrifugation through an isopycnic caesium chloride gradient. Scale bars 50 nm.

Previous negatively-stained electron microscopy (nsEM) analysis of a JC virus ΔAgno mutant revealed virions which were of a similar size to WT particles but appeared less regular or less ordered [42]. To investigate whether BK agnoprotein might also influence virion morphology, virions were purified from the media and cell lysates of WT and ΔAgno transfected cells using a modification to previously described protocols [4,43], by centrifugation in an isopycnic cesium chloride gradient. nsEM analysis of purified virions revealed polyhedral particles with a diameter of 45–50 nm (Figure 3), indistinguishable from WT BK Dunlop virions purified using the same protocol. These findings show that the inability of the ΔAgno to propagate an infection is unlikely to be due to defects in virion assembly or infectivity but more likely due to a defect(s) in virion release.

### 2.4. Agnoprotein Is Required for the Nuclear Egress of BK Particles

**Figure 4:**
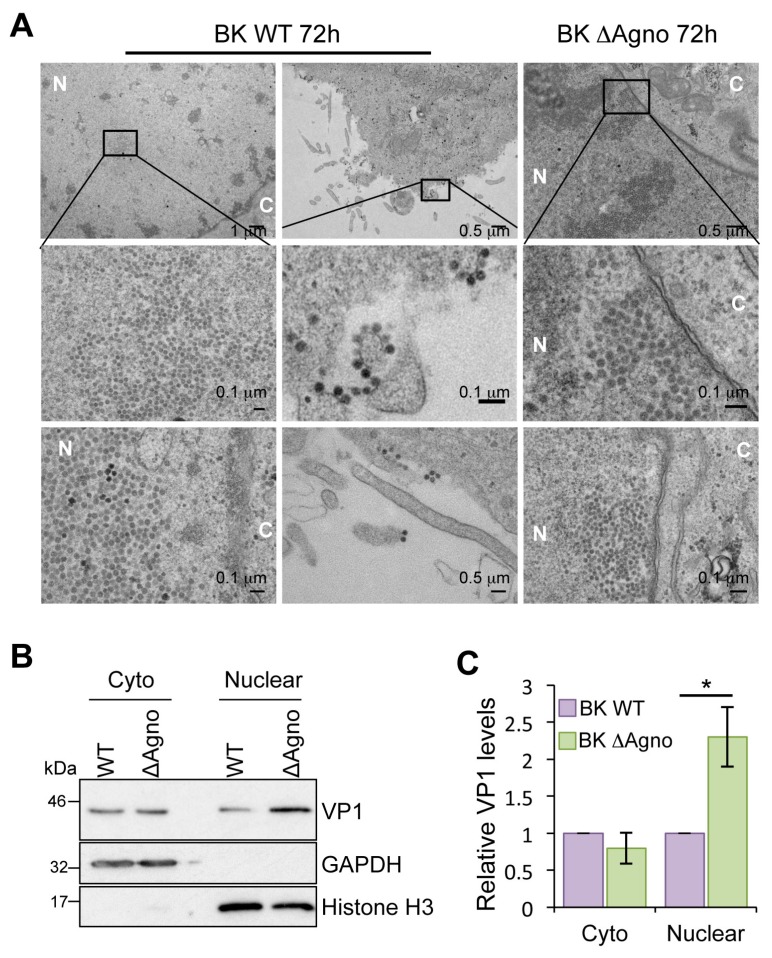
Agnoprotein facilitates nuclear release of BK virions. (**A**) Electron microscopy analysis of BK WT and ΔAgno infected RPTE cells (*n* = 40 cells). Boxed areas in the upper panel are shown at higher magnification in the middle panels. Viral particles of about 40 nm in diameter were found in the nuclei of BK WT and ΔAgno transfected cells. Nuclei (N) and cytoplasm (C) are labeled. Scale bars are shown in the panels; (**B**) Cell fractionation of RPTE cells transfected with BK WT or ΔAgno genomes. Fractions were probed with for VP1 expression. Antibodies detecting GAPDH and Histone H3 served as markers for the cytoplasm and nuclear fractions; (**C**) Quantification of the Western blot data was performed using ImageJ software (1.8.0_101, NIH, USA) on the VP1 positive bands and is represented relative to BK WT VP1. The graph corresponds to an average from three independent experimental repeats. Significance was analyzed by Student’s *t*-test and is indicated by an asterix * *p* <0.05.

The data accumulated suggested that the ΔAgno mutant was defective with regard to virion release. This prompted us to monitor the different steps of virus release at the single cell level by electron microscopy. Virions with the distinctive morphology of a polyomavirus were readily detected in nuclear, cytoplasmic and plasma membrane compartments of RPTE cells infected with WT BK virus (Figure 4). In contrast, virions were exclusively detected in the nuclei of ΔAgno infected cells. No cytoplasmic or plasma membrane localized virions were detected after the examination of numerous ∆Agno infected RPTE cells (*n* = 40), whereas at least 98% of WT BK virus infected cells had clear cytoplasmic and/or plasma membrane localized virions. We extended this analysis by performing a biochemical fractionation of RPTE cells. GAPDH and Histone H3 served as markers for the cytoplasmic and nuclear fractions, respectively. Whilst similar levels of VP1 protein were observed in both fractions in WT BK expressing cells (Figure 4B,C), in agreement with the electron microscopy data, we observed a significant enrichment for VP1 protein in the nuclear fraction of ΔAgno expressing cells. Together, these data indicate that agnoprotein is required for the release of BK virions from the nucleus of RPTE cells.

### 2.5. Agnoprotein Does Not Cause Gross Destabilization of the Nuclear Membrane

**Figure 5:**
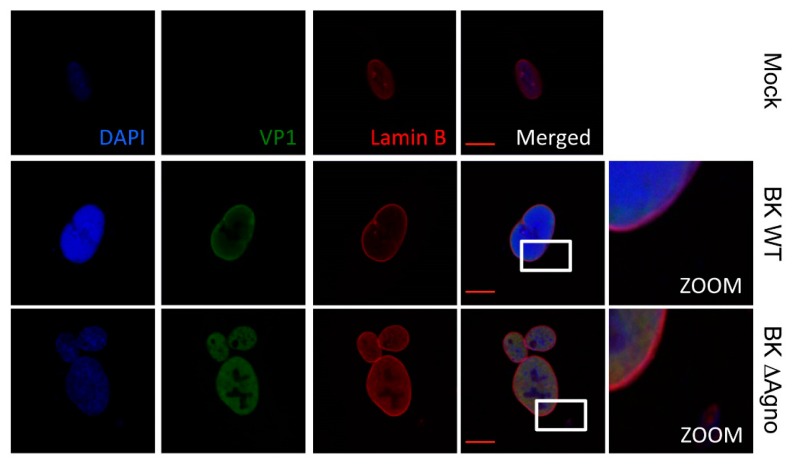
Lamin B localization is not altered by agnoprotein. Immunofluorescence staining of RPTE cells 72 h post transfection with BK WT or ΔAgno genomes. Cells were incubated with antibodies against VP1 and Lamin B and a secondary antibodies. Alexa Fluor 488 chicken anti-mouse and Alexa Fluor 594 chicken anti-rabbit. 4′,6-diamidino-2-phenylindole (DAPI) was used to indicate cell nuclei. Representative images are shown from at least three independent experimental repeats and white frames indicate area shown in the zoomed image. Scale bar 10 μm.

Exogenous expression of JC agnoprotein has been shown to uncouple interactions between proteins within the nuclear lamina, which might facilitate nuclear release of virions [44]. To investigate whether BK agnoprotein expression is also associated with a disruption of the nuclear membrane architecture, immunofluorescence microscopy was performed on markers of the nuclear membrane. Overall, staining with an antibody against Lamin B, a structural component of the inner nuclear membrane, revealed an absence of the nuclear envelope invaginations previously associated with JC agnoprotein expression [44]. Lamin B localization was unaffected in both WT and ΔAgno containing cells (Figure 5). We also noted subtle differences in the localization of VP1 expressed from WT and ΔAgno genomes. VP1 expressed in BK WT containing cells had a pronounced perinuclear localization, whereas VP1 expressed by ΔAgno appeared diffuse. Similar observations have been observed in cells infected with an SV40 agnoprotein mutant [45]. These subtle differences were not consistent between experiments so it is unclear whether they reflect a true effect of agnoprotein on VP1 localization.

### 2.6. Host α-SNAP Is Necessary for BK Egress

**Figure 6:**
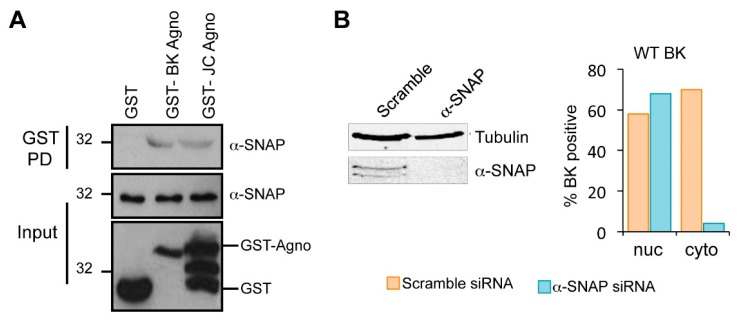
The agnoprotein binding partner α-SNAP is required for BK virion release. (**A**) Recombinant GST-agnoprotein interacts with α-SNAP. Bacterial expressed GST-agnoproteins from BK and JC virus bound to glutathione-agarose beads were incubated with RPTE cell lysates. GST alone served as a negative control. Bound samples were probed with an anti-α-SNAP antibody; (**B**) Quantification of transmission electron microscopy data. RPTE cells infected with BK WT were treated with siRNA targeting α-SNAP or a scrambled control and electron microscopy used to quantify the numbers cells demonstrating BK virions in nuclear (nuc) and cytoplasmic (cyto) compartments from 50 cells. Associated Western blots for α-SNAP to confirm effective knockdown. Tubulin serves as a loading control.

In the absence of gross perturbation of the nuclear envelope, we investigated the role of cellular proteins in the nuclear egress activity of agnoprotein. Few BK agnoprotein interacting proteins have been identified [22]. Amongst these, α-soluble N-ethylmaleimide sensitive fusion (NSF) attachment protein (α-SNAP) was of interest given its role in vesicular trafficking [46]. The interaction between agnoprotein and α-SNAP was first confirmed using recombinant glutathione S-transferase (GST)-agnoprotein proteins produced in bacteria. No interaction was seen when GST alone was incubated with mammalian cell lysates containing α-SNAP (Figure 6A). In contrast, an interaction was observed with GST-BK agnoprotein, confirming previous findings [46]. A similar interaction with α-SNAP was observed with JC virus agnoprotein, indicating that α-SNAP might be a common agnoprotein binding partner. The consequences of an interaction between BK agnoprotein and α-SNAP for the BK life cycle have not been studied. To elucidate a potential role for α-SNAP in BK virion egress, we depleted α-SNAP from RPTE cells transfected with BK genomes using a pool of validated siRNA. A scrambled siRNA served as a control for potential off target effects (Figure 6B). To determine whether α-SNAP might function within the BK egress pathway we analyzed the sub-cellular localization of BK virions in α-SNAP depleted cells. RPTE cells were infected with BK WT virus prior to transfection with α-SNAP or scramble control siRNA to avoid any potential impact of α-SNAP loss on virus entry. Cells were subsequently analyzed by electron microscopy and the presence of virions in nuclear, cytoplasmic and extracellular compartments was scored for 50 cells of each condition. In scramble siRNA transfected samples, 70% of the cells counted were positive for the presence of cytoplasmic and/or plasma membrane localized virions. In contrast, in α-SNAP depleted cells only 6% of the cells counted had detectable particles in the cytoplasm (Figure 6B).

## 3. Discussion

Despite intensive research, the mechanisms by which polyomavirus particles are released during infection remain poorly understood. It is broadly believed that as non-enveloped viruses, polyomaviruses exit from an infected cell by a process of cell lysis. However, non-specific disintegration of a cell, and release of its potentially inflammatory milieu, might be considered detrimental to the establishment of a chronic virus infection. Rather, a process of controlled virus release would be preferable to avoid immune detection. Evidence for the existence of non-lytic release of polyomaviruses exists for SV40 [47] and has recently been shown for BK [38]. Despite these observations, the role of virus proteins in the release of BK virus remains poorly described.

Here we describe the agnoprotein as a critical factor for the shuttling of progeny BK virions from the nucleus, the site of polyomavirus replication and assembly, to the cytoplasm for release. Identified nearly two decades ago, the agnoprotein is expressed by a limited number of human polyomaviruses. Studies have produced contradictory findings, confounding our understanding of the contribution of this small auxiliary protein to the life cycles of polyomaviruses. Using a BK genome containing a mutation, which converted the agnogene start codon into a stop codon, our data generated from transfected genomes or from virus infection studies demonstrates that loss of agnoprotein correlates with a reduction of virus secretion into the extracellular environment. This deficit in release resulted in reduced virus propagation and an accumulation of virions within infected cells. Transfection studies also highlighted a concomitant increase in BK transcript levels and genome replication in cells lacking agnoprotein. Whether this was due to potential negative regulation of LT function by agnoprotein, as has been reported in JC virus [32], or the consequence of an interaction with the host proliferating cell nuclear antigen (PCNA) protein [48] was not tested further.

Release of BK virus has recently been shown to be sensitive to the actions of the broad spectrum anion channel blocker DIDS [38]. In addition to cellular targets, DIDS can block the channel activity of the enterovirus 2B protein [39]. Many viruses encode small hydrophobic proteins, termed viroporins, that form pore-like structures similar to 2B [40]. JC agnoprotein is a viroporin, essential for JC virion release [28,49]. It is plausible that BK agnoprotein also performs a viroporin function to aid in virion release. Despite this possibility, addition of DIDS further reduced virion release in ΔAgno infected cells, suggesting an agnoprotein independent target for this compound. These data also imply that agnoprotein-independent egress pathways exist that must contain the cellular target of DIDS.

Whilst some studies have suggested that loss of agnoprotein impairs polyomavirus maturation and infectivity, our data clearly demonstrates that virions produced in the absence of agnoprotein are infectious and retain WT morphology. Instead, our results are consistent with the notion that loss of agnoprotein blocks the physical release of BK virions from infected cells, rather than affecting virion infectivity. These observations raised the question of where virions are localized within an infected cell in the absence of agnoprotein. We observed virions throughout the cell in BK WT infected cells, with high concentrations of virions in the nucleus but clear localization of virions in cytoplasmic compartments and at the plasma membrane. In contrast, while we could also observe high concentrations of virions in the nucleus of ΔAgno infected cells, virtually no virions were identified in the cytoplasm. Given the lack of gross impact on nuclear membrane morphology at the time-points analyzed, we reasoned that agnoprotein might recruit host factors to promote virion nuclear egress. Whilst a number of host interacting partners have been identified for JC virus agnoprotein, the BK agnoprotein interactome is less understood [22]. We focused on α-SNAP because of its critical role in vesicular trafficking [46,50,51]. α-SNAP is a known BK agnoprotein binding protein, however, its role in the virus life cycle has not been studied. Knockdown of α-SNAP conferred an agnoprotein knockout phenotype on WT BK by preventing nuclear virion egress. Importantly, loss of α-SNAP had no cumulative impact on the ΔAgno phenotype, suggesting that both proteins may function within the same egress pathway (data not shown). Whilst our data implicates BK agnoprotein and α-SNAP in virion egress, how virus is transported from the nucleus to the cytoplasm remains to be understood. A crucial area of future work will be to determine the route and mode of virion transport and to define the precise function of α-SNAP within this process. In infected polarized epithelial cells, SV40 virions have been observed within cytoplasmic membrane reticular structures, contiguous with the nuclear membrane and ER [47]. Moreover, whilst studying the effects of DIDS on the virus lifecycle, BK virions were noted in cytoplasmic vacuoles and in LAMP-1 positive vesicles, implicating the secretory system in virus release [38]. Given that α-SNAP is an integral regulator of endoplasmic reticulum to Golgi trafficking, it is tempting to speculate that BK may usurp this host secretory pathway to traffic virions from the nucleus to exterior of the cell for release. In support of this idea, treatment with the ionophore monensin impaired the release of SV40 from polarized epithelial cells and resulted in an accumulation of virions in the cytoplasmic reticular structures [47]. As part of our ongoing studies, it will also be of interest to determine whether SV40 and JC virus utilize similar processes for virion release. Loss of agnoprotein imparts an egress defect in both viruses, and JC agnoprotein is known to interact with components of the trafficking apparatus [49]. In this study we demonstrated that α-SNAP is an interacting partner for JC agnoprotein, and as such it may also be required during virus release.

In summary, our data show clearly that agnoprotein is a key virus-encoded regulator of BK virus release, and through an interaction with α-SNAP aids in an active egress pathway. Our findings provide further evidence for a virus regulated release mechanism.

## 4. Methods and Materials

### 4.1. Cell Culture

BK virus stocks were generated in Vero cells, which were maintained in Dulbeco’s Modified Eagle’s medium (DMEM) supplemented with 10% fetal calf serum (FCS) and 50 IU/mL penicillin/streptomycin. Primary renal proximal tubular epithelial (RPTE) cells (Lonza, Basel, Switzerland) were cultured in renal epithelial growth media with the REGM Bulletkit supplements (Lonza) at 37 °C with 5% CO_2_ in a humidified incubator as described [4].

### 4.2. Generation of an Agnoprotein Knockout BK Dunlop Genome

A BK knockout genome was created by site directed mutagenesis of the pGEM7-Dunlop plasmid (a gift from Michael Imperiale, University of Michigan) using the QuikChange site directed mutagenesis kit (Thermo Fisher, Waltham, MA, USA) and the primer pair ^5′^CCA GTT AAA CTG GAC AAA GGC CTA GGT TCT GCG CCA GCT GTC ACG^′3^ and ^5′^CGT GAC AGC TGG CGC AGA ACC TAG GCC TTT GTC CAG TTT AAC TGG^′3^ (Agilent Technologies, Santa Clara, CA, USA). The entire genome was subsequently sequenced to confirm the introduction of the mutation and ensure that secondary mutations had not arisen.

### 4.3. Transfection of Virus Genomes

Cells were transfected with WT BK Dunlop or ΔAgno genomes using the NanoJuice transfection kit (Merck Millipore, Darmstadt, Germany) according to the manufacturer’s instructions. The transfection mixture was removed and replaced with fresh media 8 h post-transfection.

### 4.4. Virus Culture and Purification

BK Dunlop was cultured and purified on a cesium chloride linear gradient as previously described [4]. RPTE cells were infected at approximately 50% confluency with purified virus in Opti-MEM (Thermo Fisher, Waltham, MA, USA) and incubated at 4 °C for 1 h with shaking every 15 min. Cells were subsequently transferred to 37 °C after the incubation.

### 4.5. Cell Infections and Harvesting Virus

For virus release assays, RPTE cells were infected with BK virus at 1 IU cell^−1^. After 1 h, the medium was removed, the cells gently washed in phosphate buffered saline (PBS) and then fresh medium added. For inhibitor studies, at 48 h post-infection 50–100 μM DIDS or DMSO only was added. At 72 h post infection the culture media was collected and centrifuged for 5 min at 2000× *g* to pellet any cell debris in the media, and then the supernatant transferred to new tubes. This was repeated to ensure no cell debris was present, before centrifuging the supernatant at 100,000× *g* for 2 h to pellet the virus. The media was aspirated and the pellets were resuspended in 1/20th of the original volume. The RPTE cell monolayer was harvested separately in 1 mL of REGM and freeze thawed 3-times to release cell-associated virus. Infectious virus titers in the release and cell-associated fractions were determined by fluorescence focus unit (FFU) assay [38].

### 4.6. Fluorescent Focus Unit Assay Using IncuCyte ZOOM Analysis

RPTE cells were seeded out into 96-well-plate (2 × 10^3^ cells per well, in a total volume of 100 μL) and incubated for 16 h. Purified BKPyV was serially diluted two-fold into serum-free media (in a total volume of 100 μL per well) and allowed to infect RPTE cells for 2 h at 37 °C. Infected cells were washed once with phosphate-buffered saline (PBS) and fresh media was added. RPTE cells were incubated for 48 h at 37 °C. Cells were fixed with 4% paraformaldehyde for 10 min at room temperature and washed with PBS. Fixed RPTE cells were permeabilised with 0.1% Triton-X100 (Sigma-Aldrich, St-Louis, MI, USA) in PBS, washed and incubated overnight at 4 °C in primary antibody against VP1 protein. Anti-VP1 primary antibody was used at 1:250 dilutions (in PBS with 1% BSA). Cells were further washed and incubated with a fluorophore-488-conjugated chicken anti-mouse secondary antibody (1:250 in PBS with 1% BSA) for 1 h at 37 °C. Finally, RPTE cells stored in PBS and the plate was imaged with the IncuCyte ZOOM instrument (Essen BioScience, Ann Arbor, MI, USA). The software parameters with a 10× objective were used for imaging [37]. The number of positive infected cells per well was calculated. BKPyV titer was measured by multiplying the number of positive-infected cells/well by the corresponding dilution factor and the IU mL^−1^ was determined by calculating the number of infected cells in the entire well from the mean number of infected cells in the 10 fields of view, and then the number of infectious units calculated. [37].

### 4.7. Immunofluorescence

RPTE cells (1 × 10^5^) grown on glass coverslips were fixed with 4% paraformaldehyde for 10 min. RPTE cells were then washed twice in PBS and permeabilized with 0.1% Triton X-100 for 10 min. Non-specific targets were blocked by incubation in blocking buffer (5% BSA in PBS) for 30 min. Cells were incubated with primary antibodies against VP1 (Pab597—a gift from Chris Buck, National Cancer Institute; used 1:250)) and Lamin B1 (Abcam, Cambridge, UK; ab16048) overnight at 4 °C. Cells were washed three times in PBS prior to incubation in secondary antibodies Alexa Fluor 488 chicken anti-mouse and Alexa Fluor 594 chicken anti-rabbit (Invitrogen, Carlsbad, CA, USA) for 1 h at room temperature. Cells were washed three times in PBS prior to mounting onto microscope slides using Prolong Gold Antifade Reagent with DAPI (Thermo Fisher, Waltham, MA, USA). Samples were observed under a Zeiss LSM 700 laser (Carl Zeiss Ltd, Jena, Germany) scanning confocal microscope under an oil-immersion objective lens.

### 4.8. Western Blotting

Triton lysis buffer (10 mM Tris (pH 7.6), 10 mM sodium phosphate, 130 mM NaCl, 1% Triton X-100, 20 mM N-ethylmaleimide, complete protease inhibitor cocktail; Roche, Basel, Switzerland) was used to harvest total cellular protein from the infected cells. Protein concentration was quantified with the Bradford assay (Bio-Rad, Hercules, CA, USA). Lysates were separated by SDS PAGE and following transfer to nitrocellulose membrane were probed with the following antibodies diluted in 5% non-fat dried milk in TBS with 0.1% Tween-20; mouse anti-VP1 pAb-597 (1:5000), rabbit anti-VP2/VP3 (Abcam; ab53983; used 1:1000), mouse anti-Large T antigen (Abcam; ab16879; used at 1:200) and α-SNAP (Santa Cruz 4E4); used at 1:1000) and mouse anti-GAPDH (Santa Cruz, Dallas, TX, USA; used 1:5000).

### 4.9. Quantitative PCR

Total DNA was extracted from infected cells using the E.Z.N.A. Tissue DNA kit (Omega Bio-Tek, Norcross, GE, USA) and 10 ng of DNA was analysed by qPCR using the Quantifast SYBR Green PCR kit (Qiagen, Venlo, The Netherlands) with the following primers against BK Dunlop; BK Forward ^5′^TGT GAT TGG GAT TCA GTG CT^′3^ and Reverse ^5′^AAG GAA AGG CTG GAT TCT GA^′3^. To extract DNA from released virus, the culture media was collected and centrifuged for 5 min at 2000× *g* to pellet any cell debris in the media, and then the supernatant transferred to new tubes. This was repeated to ensure no cell debris was present, before centrifuging the supernatant at 100,000× *g* for 2 h to pellet the virus. Virus was treated with RQ1 RNase-free DNase (Promega, Madison, WI, USA) for 30 min at 37 °C to remove any unprotected DNA, and the reaction terminated by the addition of DNase Stop Solution and incubation for 10 min at 65 °C. A serial dilution of the pGEM7-Dunlop plasmid was used to calculate the copy number per microgram of DNA.

### 4.10. Quantitative Reverse Transcriptase PCR

Total RNA was extracted from RPTE cells using the E.Z.N.A Total RNA Kit I (Omega Bio-Tek) following the manufacture’s protocol. One μg of the total extracted RNA was reverse transcribed using the iScript^TM^ cDNA Synthesis Kit (Bio-Rad) based on the protocol instructions. Quantitative Real-time PCR was performed using the QuantiFast SYBR Green PCR kit (Qiagen) and specific primers against VP1. The PCR reaction was carried out on a Corbett Rotor-Gene 6000 (Qiagen) following three different steps. The initial activation step for 10 min at 95 °C and a three-step cycle of denaturation of 10 s at 95 °C; the second step of annealing for 15 s at 60 °C and the step of extension for 20 s at 72 °C. All the three different steps were repeated 40 times and concluded by melting curve analysis. U6 was used as normaliser control.

### 4.11. Electron Microscopy

Negative staining of BK virus particles was carried out as follows, 3.5 μL aliquots of purified wild-type BK or ΔAgno in buffer A were applied to continuous carbon grids that had been glow-discharged for ~30 s in air using a PELCO easiGlow™ (Ted Pella Inc, Redding, CA, USA). The samples were then stained with 1% uranyl acetate solution before being allowed to dry in air for 5 min. Samples were imaged on a Tecnai G^2^-Spirit transmission EM (FEI, Eindhoven, The Netherlands) at 120 keV, equipped with a Gatan US1000XP CCD camera (Gatan, Pleasanton, CA, USA). Images of virions were recorded at 30,000× magnification.

### 4.12. Transmission Electron Microscopy in Cells

Cells were fixed in 0.5% glutaraldehyde in 200 mM sodium cacodylate buffer for 30 min, washed in buffer and secondarily fixed in reduced 1% osmium tetroxide, 1.5% potassium ferricyanide for 60 min. The samples were washed in distilled water and stained overnight at 4 °C in 0.5% magnesium uranyl acetate, washed in distilled water and dehydrated in graded ethanol. The samples were then embedded flat in the dish in Epon resin. Resin filled stubs were placed on embedded cell monolayers and polymerized. Ultrathin sections (typically 50–70 nm) were cut parallel to the dish and examined in a FEI Tecnai20 electron microscope (FEI Co., Eindhoven, The Netherlands) with CCD camera image acquisition.

### 4.13. Cell Fractionation

RPTE (2 × 10^5^) cells seeded in 6 well plates were scraped into PBS. Cell pellets were resuspended in 100 μL cytoplasmic lysis buffer (100 mM Tris pH 7.5, 100 mM NaCl, 5 mM MgCl_2_, 0.5% NP-40, complete protease inhibitor cocktail (Roche)) and lysed on ice for 20 min. Then samples were centrifuged at 9600× *g* for 20 min at 4 °C. The supernatant, containing the cytoplasmic fraction, was collected and the pellet washed twice in cytoplasmic lysis buffer and then resuspended in 100 μL RIPA buffer (50 mM Tris pH 7.5, 150 mM NaCl, 1% NP-40, 0.5% sodium deoxycholate, 0.1% SDS, protease inhibitors). Samples were centrifuged at 9600× *g* for 10 min at 4 °C and the nuclear fraction supernatant collected.
